# Revisiting the Key Driving Processes of the Decadal Trend of Aerosol Acidity in the U.S

**DOI:** 10.1021/acsenvironau.1c00055

**Published:** 2022-05-06

**Authors:** Guangjie Zheng, Hang Su, Yafang Cheng

**Affiliations:** † Minerva Research Group, Max Planck Institute for Chemistry, Mainz 55128, Germany; ‡ Multiphase Chemistry Department, Max Planck Institute for Chemistry, Mainz 55128, Germany

**Keywords:** aerosol acidity, multiphase buffer system, driving processes, long-term trend, organic acids buffering, ammonium-to-sulfate ratio, non-volatile cations

## Abstract

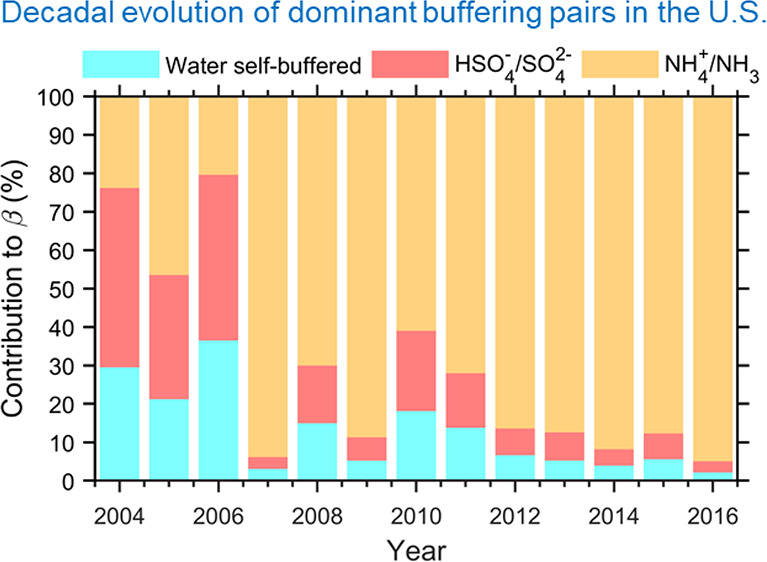

Acidity is one essential parameter in determining the aqueous phase physical and chemical processes in the atmosphere and strongly influences the climate, ecological, and health effects of aerosols. Traditionally, aerosol acidity is thought to increase with emissions of atmospheric acidic substances (SO_2_, NOx, etc.) and decrease with that of alkaline ones (NH_3_, dust, etc.). However, decade-long observations in southeastern U.S. seem to disagree with this hypothesis: while the emissions of NH_3_ versus SO_2_ enhanced by over three times, the predicted aerosol acidity is stable, and the observed particle-phase ammonium-to-sulfate ratio is even decreasing. Here, we investigated into this issue with the recently proposed multiphase buffer theory. We show that historically, there is a transition in the dominant drivers of aerosol acidity in this region. Under the ammonia-poor conditions before ∼2008, the acidity is governed by HSO_4_^–^/SO_4_^2–^ buffering and the water self-buffering effect. Under the ammonia-rich conditions after ∼2008, aerosol acidity is mainly buffered by NH_4_^+^/NH_3_. Buffering from the organic acids is negligible in the investigated period. In addition, the observed decrease in ammonium-to-sulfate ratio is due to the increased importance of non-volatile cations, especially after ∼2014. We predict that until ∼2050, the aerosols will remain in the ammonia-buffered regime, and the nitrate will remain largely (>98%) in the gas phase in southeastern U.S.

## 1 Introduction

Aerosol acidity is one central parameter in atmospheric research, which largely regulated the thermodynamics and chemical kinetics in atmospheric multiphase chemistry,^1−4^ therefore influencing the effects of aerosols on health, ecosystem, and climate.^3,5−9^ Traditionally, aerosol acidity is thought to increase with emissions of atmospheric acidic substances (SO_2_, NOx, etc.) and decrease with that of alkaline ones (NH_3_, Na^+^, Ca^2+^, K^+^, Mg^2+^, etc.).^2,10−16^ However, analysis of the long-term trend of aerosol acidity in southeastern U.S. (SE-US) is against this hypothesis. Over the past two decades, sulfate in southeastern U.S. has decreased by 70%, while the gas-phase ammonia concentration shows a constant or even slowly increasing trend.^10,16,17^ This is expected to result in an increase of both pH and the ammonium-to-sulfate ratio in the particle phase.^3^ In contrast, thermodynamic models predict a small change of pH varying between ∼0 and 2, while the observed ammonium-to-sulfate ratio even decreased slightly.^10^ In this sense, the U.S. aerosols behave like a “buffered system” that resists pH changes upon addition of acids or bases within a certain range.

The pioneer study of Weber et al.^10^ tried to explain the above counterintuitive phenomenon with a concept model, referred to as the W16 model hereinafter. This model assumes that (i) the resistance of U.S. aerosol pH changes upon changing ammonia/sulfur emissions is due to the (NH_4_)_2_SO_4_-NH_4_HSO_4_ transition in the aerosol water, where the relative ratio of (NH_4_)_2_SO_4_/NH_4_HSO_4_ is regulated through the partitioning of ammonia between gas and particle phase; and (ii) the observed decrease in ammonium-to-sulfate ratio is due to the limited available partitionable ammonium. However, this concept model is ambiguous in the following points. First, the governing factors of (NH_4_)_2_SO_4_-NH_4_HSO_4_ transition remain unclear. Second, the regulation effect of ammonia partitioning requires the HSO_4_^–^/SO_4_^2–^ transition. Therefore, it cannot explain the similar “buffering” effect observed in other places with higher pH levels, especially when aerosol compositions are dominated by NH_4_NO_3_. Third, the explanation for decreasing ammonium-to-sulfate ratio of limited ammonium is against the equilibrium law (i.e., Le Chatelier’s principle; see Section 5).

The recently proposed multiphase buffer theory^1^ provided new insights into these issues. Here, we revisited the determinants of the U.S. aerosol acidity trend with this theory. We found that (i) under ammonia-rich conditions, the resistance of pH changes is mainly due to the buffering effect of NH_4_^+^/NH_3_, not (NH_4_)_2_SO_4_-NH_4_HSO_4_ transition; (ii) under ammonia-poor or highly acidic conditions, the water self-buffering effect is also an important factor that resist the aerosol pH from dropping below 0, in addition to the HSO_4_^–^/SO_4_^2–^ buffering effect; and (iii) the observed decrease in ammonium-to-sulfate ratio is actually due to the increased importance of non-volatile cations (NVCs, mainly Na^+^, Ca^2+^, K^+^, Mg^2+^), not the limited availability of partitionable ammonium.^10^ Based on the projected emissions until ∼2050 in southeastern U.S., aerosol acidity is predicted to remain in ammonia buffered regime and increase only slightly (from 1 to below 2), in which pH ranges the nitrate will remain almost all (>98%) in the gas phase.

## 2 Methods

Long-term observations of aerosol compositions and gas species are conducted at the Centreville site (CTR, 32.902°N, 87.250 °W, altitude 126 m; AL, U.S.A.), as part of the Southeastern Aerosol Research and Characterization (SEARCH) network. Detailed site and instrumentation information are documented elsewhere.^18−20^ The summer (June to August) data during 2004 to 2016 are analyzed here. The PM_2.5_ chemical compositions, gas-phase ammonia mixing ratios, and meteorological parameters are used in this study.

The aerosol acidity is defined as the free molality of protons,^2,21^ which is estimated by thermodynamic models of the ISORROPIA v2.3 (ref (22)) and the E-AIM (model IV; http://www.aim.env.uea.ac.uk/aim/aim.php; last access: April 23, 2022).^23−25^ The E-AIM model is usually considered as the “benchmark” thermodynamic models,^2^ and its results are used to examine the potential influences of thermodynamic models applied on the identified driving factors. The ISORROPIA model is used as it can consider the influences of Ca^2+^, K^+^, and Mg^2+^, which will be discussed in Section 5. In addition, results from ISORROPIA are directly comparable with the pioneering study of Weber et al.^10^ The ISORROPIA model is run in forward mode and with the metastable assumption, and the predicted ammonia partitioning agreed well with the observations (Figure S1). Calculations of multiphase buffer capacity and the treatment of non-ideality are detailed elsewhere^1,21^ and are briefly explained where needed.

## 3 Role of Ammonia: Regulating (NH_4_)_2_SO_4_/NH_4_HSO_4_ Ratios vs Multiphase Buffering

### 3.1 W16 Model

The W16 concept model proposed that the gas–particle partitioning of NH_3_ would regulate (NH_4_)_2_SO_4_/NH_4_HSO_4_ ratios in the aerosol phase, which will thereby constrain the aerosol pH between ∼0 and 3, as the pH of pure aqueous NH_4_HSO_4_ aerosols is around 0 while that of pure aqueous (NH_4_)_2_SO_4_ aerosols is around 3 (ref (10)). This process can be represented as follows:
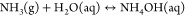
1a
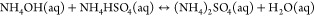
1b

**Figure 1:**
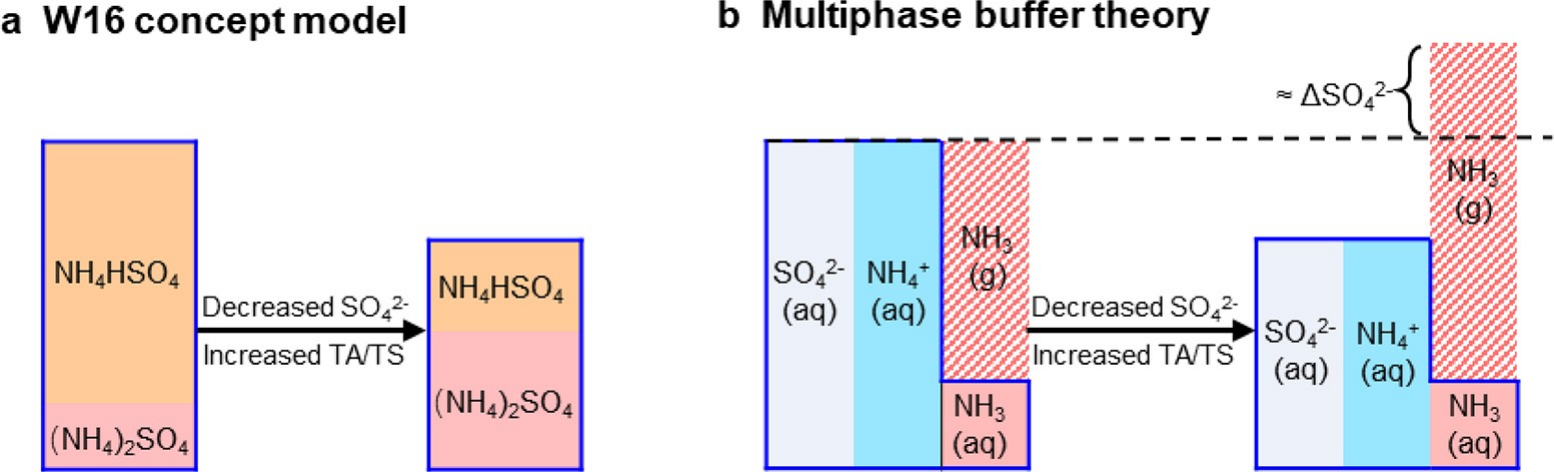
Comparison of multiphase buffer theory and the pioneering W16 concept model in explaining aerosol acidity variations in SE-US. Over the last decade in SE-US, SO_4_^2–^ has decreased substantially while the total ammonia is roughly constant, and thus the TA/TS ratios have increased. However, the pH is roughly the same. (a) W16 concept model attributed this stable pH to the conversions in (NH_4_)_2_SO_4_/NH_4_HSO_4_ ratios,^10^ while (b) multiphase buffer theory explained it as the multiphase NH_4_^+^/NH_3_ buffering effect.^1^ Note that the amount of sulfate shown in (b) indicate the charge equivalent concentrations.

In this model, the aerosol pH is regulated by the specific compounds of (NH_4_)_2_SO_4_/NH_4_HSO_4_ and is essentially a neutralization process (Figure 1a): when sulfate is decreased and the total ammonia (TA) to total sulfate (TS) ratio^3^ is increased, the increased availability of partitionable ammonia works as additional bases (NH_4_OH) into the aerosol phase, which combined with the acidic substance (NH_4_HSO_4_) to form the neutralized salt (i.e., (NH_4_)_2_SO_4_) and water. Therefore, the W16 model is in principle a neutralization model, where the aerosol pH is regulated by the degree of aqueous phase neutralization; see Weber et al.^10^ for more details about this concept model.

### 3.2 Multiphase Buffer Theory

The recently proposed multiphase buffering theory provided a new insight into the role of ammonia in regulating aerosol pH in that NH_4_^+^(aq)/NH_3_(g) works as a buffering pair, keeping aerosol pH at a certain level (around its peak buffer pH) (ref (1)). The buffering agents are conjugate acid/base pairs that differ only by one proton, which can partially absorb the added H^+^ or OH^–^ through dissociation equilibrium. The decrease of sulfate works like removing strong acids from the multiphase system; therefore, H^+^ will decrease while OH^–^ will increase. Some of the increased OH^–^ would be converted to NH_4_OH through the following equilibria (Figure 1b):
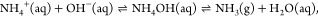
2awith the corresponding effective acid dissociation constant, *K*_a_*, being
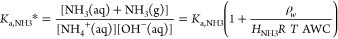
2bwhere *K*_a,NH3_ is the acid dissociation constant of ammonia in bulk aqueous phase, AWC is the aerosol water content, ρ_w_ is the water density, *T* is the temperature, *H*_NH3_ is Henry’s constant for NH_3_, *R* is the gas constant, and [NH_3_(g)] is the equivalent molality (mol kg^–1^ water) of NH_3_(g) defined as^1^
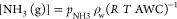
2cwhere *p*_NH3_ is the partial pressure of NH_3_ in atm.

The additional terms of *K*_a_* compared to *K*_a_ represent the influence of gas–particle partitioning. In multiphase systems, the formed NH_4_OH(aq) can volatilize into the gas phase, reducing NH_4_OH molality, further promoting the conversion of OH^–^ into NH_4_OH; see Zheng et al.^1^ for more details about the multiphase buffer theory.

Although Weber et al.^10^ pointed out the importance of gas–particle partitioning of NH_3_ in regulating aerosol pH through shifting (NH_4_)_2_SO_4_/NH_4_HSO_4_ in aqueous phase, compared with their model, the major advance of multiphase buffer theory lies in the following aspects. First, it revealed that ammonia works as the buffering agent through dissociation equilibrium, with the *K*_a_* largely controlled by AWC at a given temperature. Therefore, its buffering pH ranges, p*K*_a_* ± 1, depend weakly on the anions it is associated with (e.g., HSO_4_^–^ or SO_4_^2–^ or NO_3_^–^) assuming that AWC is the same.^21^ In comparison, the W16 model emphasized the importance of (NH_4_)_2_SO_4_-NH_4_HSO_4_ transition, which are the major forms of NH_4_^+^(aq) in SE-US; but on the other hand, it limited the application of W16 in explaining the buffering effects in other regions where the sulfate is fully neutralized into (NH_4_)_2_SO_4_ and NH_4_HSO_4_ is negligible, such as the ammonia-rich periods in northern China or western Europe. Second, the multiphase buffer theory pointed out the governing factors of aerosol pH quantitatively, with an emphasis on the dominate role of AWC and temperature in the ammonia buffered region, which is new to the W16 model.

## 4 Relative Importance of Ammonia under Different Regimes

### 4.1 Contributors of Multiphase Buffering Capacity

Resistance of pH changes of a multiphase buffer system can be represented by the buffering capacity β as follows:^1,21^
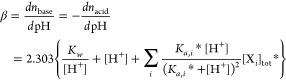
3where *n*_acid_ or *n*_base_ is the amount of acid or base added to the system in mol kg^–1^, *K*_w_ is the water dissociation constant, and X_i_ is a given buffering agent. Contribution of X_i_ to total β, β_i_, is determined by *K*_a,i_* and [X_*i*_]_tot_*. *K*_a,i_* is the effective acid dissociation constant that determines the buffering pH range of X_i_, while [X_*i*_]_tot_* is the total equivalent molality of X_*i*_ including those existing in the gas phase, as the gas–particle partitioning also plays a role (Figure 1). [X_*i*_]_tot_* determines the maximum buffer capacity X_*i*_, which is found at pH = p*K*_a,i_*. Note that the first two terms in eq 3 ([H^+^] and *K*_w_[H^+^]^−1^) represent the water self-buffering effect, which is the intrinsic inertia of water against pH changes at highly acidic or alkaline (high [OH^–^]) conditions. This effect arose from the linear-log scale relationship of [H^+^] or [OH^–^] and pH, which exists even for a non-buffered system.

### 4.2 Changes in Major Buffer Capacity Contributors

**Figure 2:**
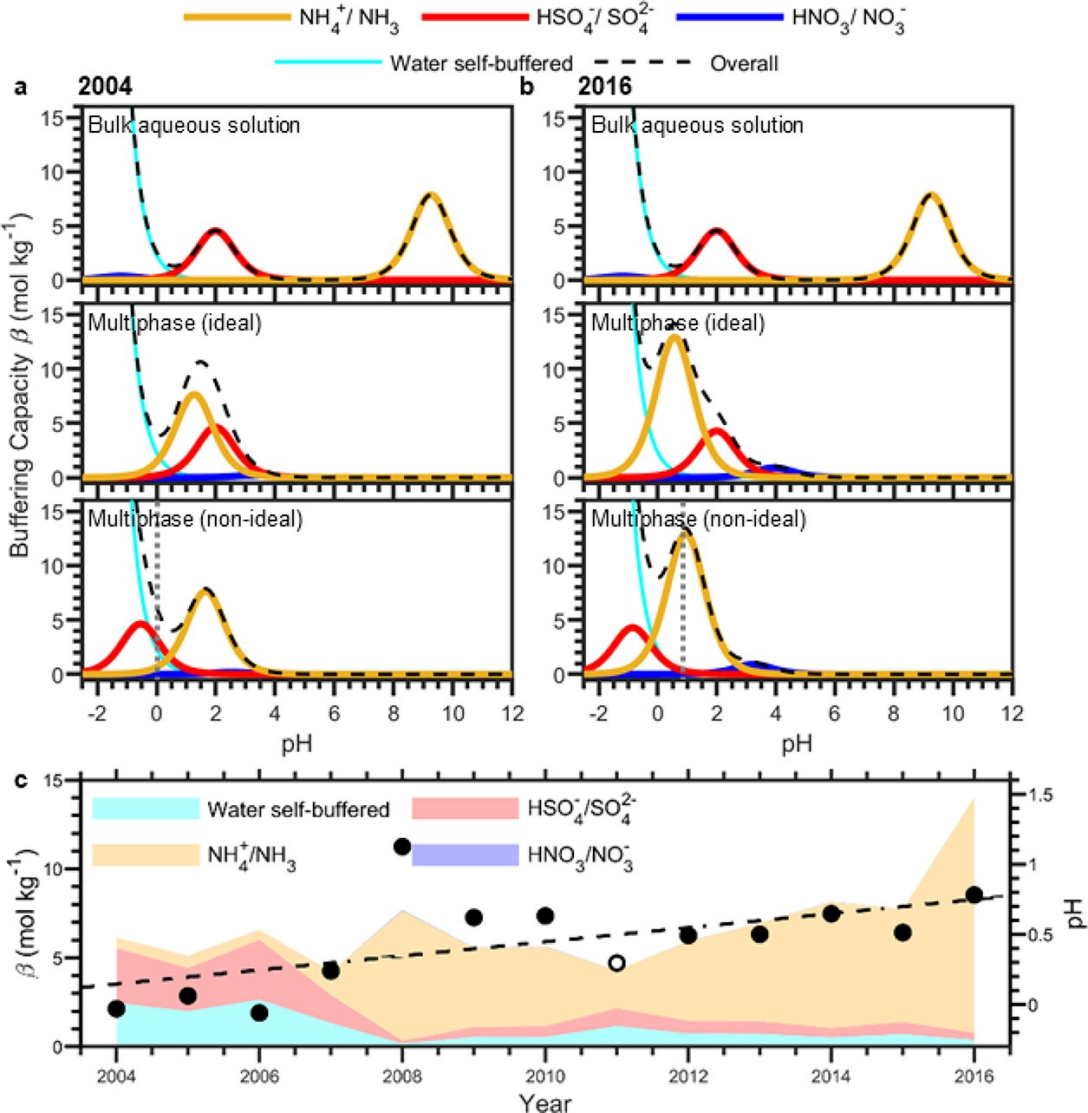
Buffering capacity curve for US scenario. The inputs are based on average summertime SEARCH data at the CTR site. Here, the influence of NVCs is not considered (see Section 5), while NH_3_(g) is included. In panel (c), the left *Y* axis correspond to β shown by the shaded areas, while the right *Y* axis correspond to the pH shown by the filled black circles. As the NH_3_(g) measurements are missing in 2011, it was assumed to be the average of NH_3_(g) in 2010 and 2012 (the hollow circle in Figure 2c). The black dashed line indicates the fitted annual pH trends for reference. The result shown here is based on the ISORROPIA model, while that based on the E-AIM model is shown in Figure S2. Although the detailed pH values predicted by these two models can differ by ±0.3 units, both models indicate the same variations in the dominant buffering regimes. Note that the pH jump in 2008 is likely related to minor bugs in the ISORROPIA algorithm (see Figure S3).

Here, we reanalyzed the historical trend of U.S. aerosol acidity by the multiphase buffer theory, with a focus on identifying the contribution of individual drivers. For illustration, buffering capacity curves of southeastern U.S. aerosols are calculated under past (summer 2004) and current (summer 2016) conditions (Figure 2), based on measurement at the SEARCH-CTR site. Data from the same measurement site has been used in the analyses in Weber et al.^10^ but only until 2013 (Section 2). Here, the results are based on the ISORROPIA model, the same as in Weber et al.;^10^ while results estimated by the E-AIM model lead to the same conclusions (Figure S2). Judging from the average TA/TS ratios, the 2004 and 2016 scenarios are ammonia-poor (TA/TS < 2) and ammonia-rich (TA/TS > 2) conditions, respectively.^3^ Major buffering agents considered here are HNO_3_(g + aq)/NO_3_^–^(aq), HSO_4_^–^(aq)/SO_4_^2–^(aq), and NH_3_(g + aq)/NH_4_^+^(aq).

For both scenarios, the buffering ranges of these species are quite different from that under the bulk conditions (Figure 2a,b). Abundances of total HNO_3_ is too low to offer any efficient buffering. Although being a weak base in bulk solutions, NH_3_(g + aq)/NH_4_ + (aq) is generally buffering in the range of 0–3, determined by the AWC concentrations (eq 2). Non-ideality elevated its buffering range only slightly (∼0.4).^21^ In comparison, the non-volatile HSO_4_^–^(aq)/SO_4_^2–^(aq) pair is not influenced by AWC but is strongly influenced by the non-ideality due to the larger sensitivity of their activity coefficients.^21^ Its p*K*_a_ moved from ∼2 under ideal conditions to −0.9 to −0.5 considering the non-ideality.

Average compositions of 2004 is in the ammonia-poor conditions (TA/TS = 1.65), and the sulfate cannot be fully neutralized into (NH_4_)_2_SO_4_ (ref (3)). At the predicted pH of ∼0, β is dominated by water self-buffering effect and HSO_4_^–^/SO_4_^2–^, while ammonia plays a minor role. In contrast, in 2016 (Figure 2b) when the aerosols are ammonia-rich (TA/TS = 3), β is solely dominated by NH_3_(g + aq)/NH_4_ + (aq) at the predicted pH levels of ∼0.8.

### 4.3 Smooth pH Transition Despite Regime Transitions

Analysis of the long-term trends of SE-US acidity (Figure 2c) shows a shift in the dominant β contributor from the water–HSO_4_^–^/SO_4_^2–^ regime to the NH_4_^+^/NH_3_ regime around 2008, consistent with the transition of ammonia-poor to ammonia-rich conditions. Interestingly, the pH changes are smooth despite the regime transitions. This is mainly due to the low AWC levels in SE-US, which renders the NH_4_^+^/NH_3_ buffering pH ranges (0–3) adjacent to that of [H^+^] and HSO_4_^–^/SO_4_^2–^. Therefore, the system β is constantly high over a wide range of pH < 3 (Figure 2a,b). This is also the reason as to why calculating the pH with/without NH_3_(g) would result in a small difference in predicted pH (∼ 1 unit) in the U.S.^10,26,27^ In regions with higher aerosol mass concentrations, the patterns can be quite different.^1^

### 4.4 Potential Contributions of Organic Acids to Buffering Capacity

**Figure 3:**
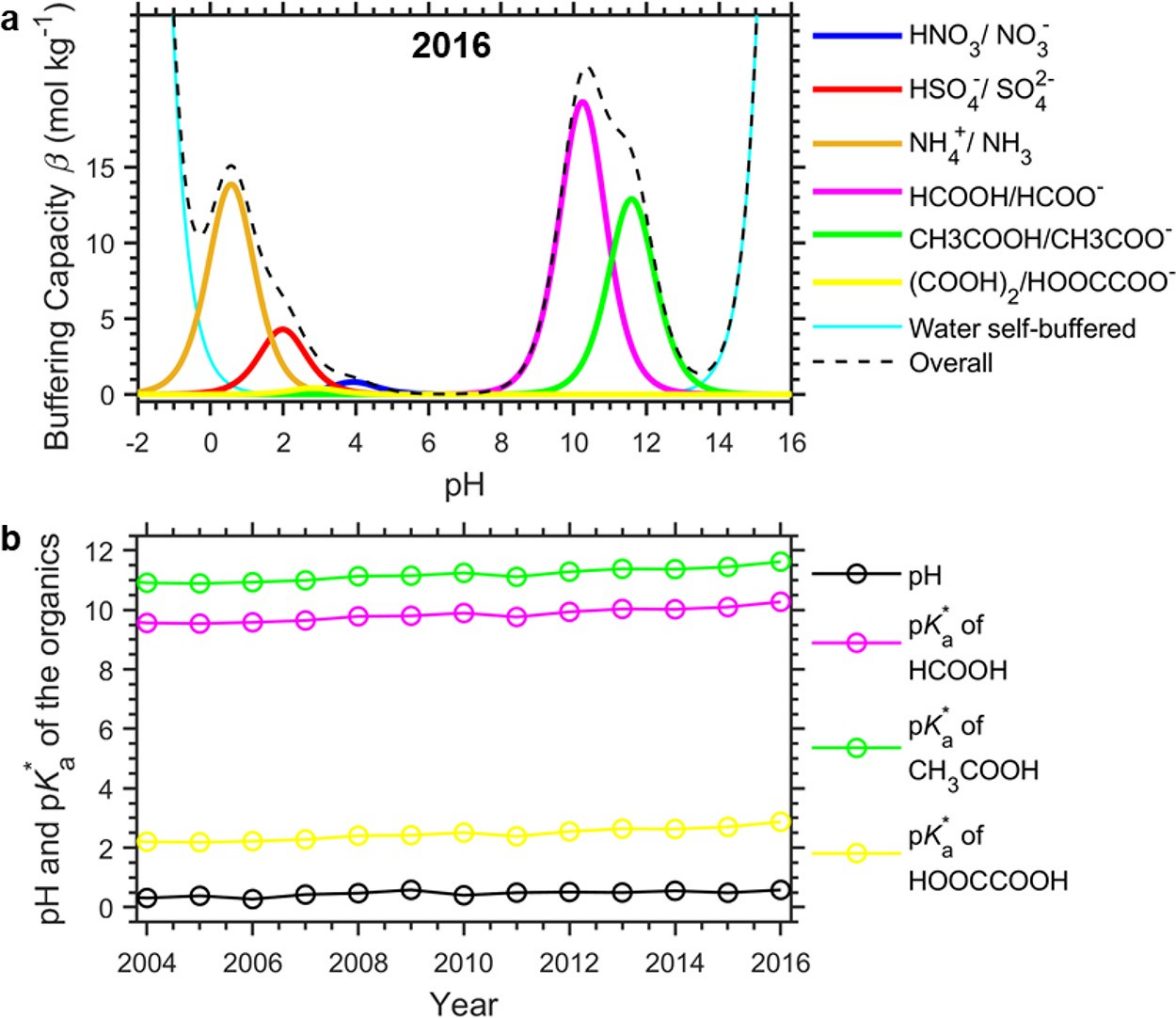
Potential influence of organic acids in the buffer capacities in SE-US. (a) Example buffering capacity curve based on the average summer 2016 conditions at the CTR site. (b) Annual trends in ISORROPIA-predicted pH and ideal p*K*_a_* of major organic acids in summer SE-US. The total (gas + particle) concentrations of the three investigated organic acids, HCOOH, CH_3_COOH, and (COOH)_2_, are based on the observation in an agriculturally intensive rural SE-US site in fall 2016,^28^ which is near the CTR site. These concentrations are expected to be satisfactory as an order-of-magnitude estimation of the concentrations in the summer CTR site.

Organic acids can also serve as the buffering agents, and their potential importance needs to be addressed given the high emissions of biogenic volatile organic compounds (BVOCs) in SE-US. Here, we examined the potential contributions of organic acids to the system buffering capacity following the method outlined in Supporting Information Section S7 and Figure S12 of Zheng et al.^1^ Three dominant organic acids, HCOOH, CH_3_COOH, and (COOH)_2_, are investigated based on the observation in fall 2016 in an agriculturally intensive rural SE-US site,^28^ which is near the CTR site. For a first-order estimation, we assume that the concentrations of these acids are constant within the study period. As shown in Figure 3a, in 2016 when the inorganic concentrations are low, the maximum buffer capacity of HCOOH and CH_3_COOH can be comparable with that of ammonia. However, they are both buffering in the alkaline pH ranges of >9. At the actual aerosol pH ranges of 0–2, their contribution to the buffer capacity is negligible. This large gaps in the p*K*_a_* and pH are always present in SE-US (Figure 3b). In comparison, while oxalic acid (COOH)_2_ can buffer at pH ∼3, its concentrations are too low to be important (Figure 3a). Therefore, the contributions of organic acids to the buffering capacity are negligible in summer SE-US.

## 5 Variations of Ammonia-to-Sulfate Ratios: Importance of NVCs

**Figure 4:**
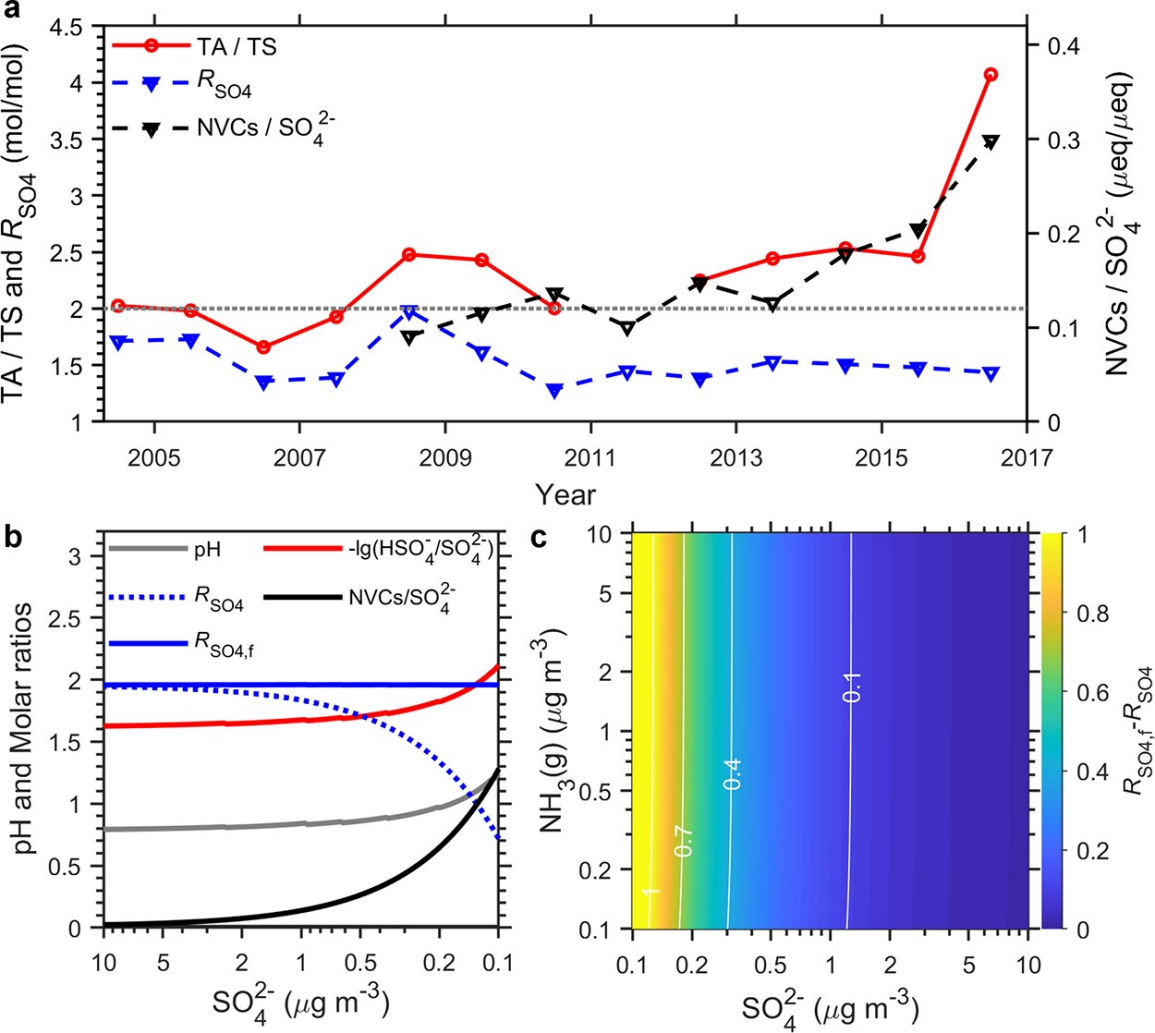
Explanations for the decreasing trend of ammonium-to-sulfate molar ratios *R*_SO4_, where *R*_SO4_ = ([NH_4_^+^] – [NO_3_^–^])/[SO_4_^2–^]_tot_. (a) Observed trend of TA/TS, *R*_SO4_, and NVCs/TS in SEARCH-CTR site in summer 2004 to 2016. (b) Simulated trend with decreasing SO_4_^2–^, assuming constant NH_3_(g) of 0.23 μg m^–**3**^ (decadal mean of CTR site). (c) Simulated variation of the difference between the corrected ratios, *R*_SO4,f_, and *R*_SO4_ with sulfate and gas-phase NH_3_. Simulation in panels (b) and (c) reproduced settings in Figure 2 of Weber *et al.*^10^, i.e., assuming a constant Na^+^ = 0.03 μg m^–**3**^, total HNO_3_ = 0.08 μg m^–**3**^, total HCl = 0.02 μg m^–**3**^, temperature of 298 K, and RH at 73.8%.

Besides the relatively stable pH, another puzzling effect of the U.S. acidity is the unexpected decrease in aerosol ammonium-to-sulfate molar ratios (*R*_SO4_) (Figure 4a). From 2004 to 2016, the aerosols in SE-US turned from ammonia-poor (TA/TS < 2) to ammonia-rich (TA/TS > 2), while the observed *R*_SO4_ decreased from 1.8 to 1.4. This disagrees with the theoretical pattern that in a H_2_SO_4_–HNO_3_–NH_3_ system, *R*_SO4_ should monotonically increase approaching 2 (i.e., when sulfate is fully neutralized) with increasing TA/TS.^29,30^

Weber et al.^10^ attributed this “counterintuitive” phenomenon to the limited available neutralizing ammonia according to their concept model. That is, at lower SO_4_^2–^, there is less available ammonium, thus a larger relative loss of NH_4_^+^ when establishing equilibrium with NH_3_(g). The ISORROPIA thermodynamic model simulations seemed to support this assumption, which showed decreasing *R*_SO4_ with decreasing SO_4_^2–^ (Figure 4b). However, the model shows simultaneously increases in HSO_4_^–^/SO_4_^2–^ and pH (Figure 4b), which is self-contradictory. Based on the W16 model, as NH_4_^+^ volatilizes and *R*_SO4_ decreases, the (NH_4_)_2_SO_4_/NH_4_HSO_4_ ratio should also decrease, and so does pH (Figure 1). Even if we consider the HSO_4_^–^/SO_4_^2–^ buffering effect, which would partially weaken the extent of pH decrease, it cannot be completely offset – well known as the equilibrium law (or Le Chatelier’s principle).^29,30^ Therefore, the larger relative loss of NH_4_^+^ must result in a simultaneous *R*_SO4_ and pH decrease, which is against their modeling result (Figure 4b). In addition, predicted changes in NO_3_^–^ are negligible and cannot explain the changes in *R*_SO4_.

Here, we re-examined the model simulations and observations and found that the decreased *R*_SO4_ is due to the increasing importance of NVCs. As the NVCs could neutralize part of the sulfate before it can be neutralized by the volatile NH_3_, the “free” total sulfate (TS_f_ = TS – 0.5NVCs) to participate in the H_2_SO_4_–HNO_3_–NH_3_ equilibrium is reduced. Therefore, both TA/TS and *R*_SO4_ would underestimate the neutralization degrees. Replacing TS with TS_f_ in the system gives the corrected definition of the ammonium to “free” sulfate ratios, *R*_SO4,f_, as
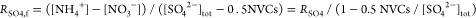
4which should be used in conditions with large NVCs/[SO_4_^2–^]_tot_ ratios.

The decreased *R*_SO4_ with increasing TA/TS as shown in both observation and simulation results can be explained with this corrected definition of eq 4. As the NVCs are assumed constant, decreasing SO_4_^2–^ will increase NVCs/TS, thereby enlarging gaps between *R*_SO4_ and *R*_SO4,f_ (Figure 4b). While *R*_SO4,f_ is kept around 2 all the time (not shown but investigated around all SO_4_^2–^ and NH_3_(g) ranges), *R*_SO4_ would decrease substantially as the system transitioned from the H_2_SO_4_–HNO_3_–NH_3_ system to a H_2_SO_4_–HNO_3_–NH_3_–NVC system, and so would their differences (i.e., *R*_SO4f_ – *R*_SO4_; Figure 4c). The observed gap between TA/TS and *R*_SO4_ also correspond to the increased NVCs/SO_4_^2–^ in recent years (Figure 4a). The mixing states may also change the gas–particle partitioning of NH_3_ and HNO_3_ and therefore the *R*_SO4_ under given TA/TS conditions. We performed a test with the fully external mixture assumption, and the difference in *R*_SO4_ is small (Figure S4).

## 6 Implications

Our finding suggested that the U.S. acidity have undergone the following three regimes: (i) the ammonia-poor conditions before ∼2008, with high acidity and therefore intrinsic stable pH levels; (ii) the NH_3_/NH_4_^+^ buffered H_2_SO_4_–HNO_3_–NH_3_ system (∼2006 to ∼2014), where TA/TS and *R*_SO4_ can be used as the indicator of NH_3_ richness and the particle phase sulfate neutralization degrees; and (iii) the NH_3_/NH_4_^+^ buffered H_2_SO_4_–HNO_3_–NH_3_–NVC system after ∼2014, where contribution from NVCs cannot be ignored but still cannot fully neutralize sulfate (i.e., NVCs/TS < 2) and *R*_SO4_ is not representative of the sulfate neutralization degrees.

**Figure 5:**
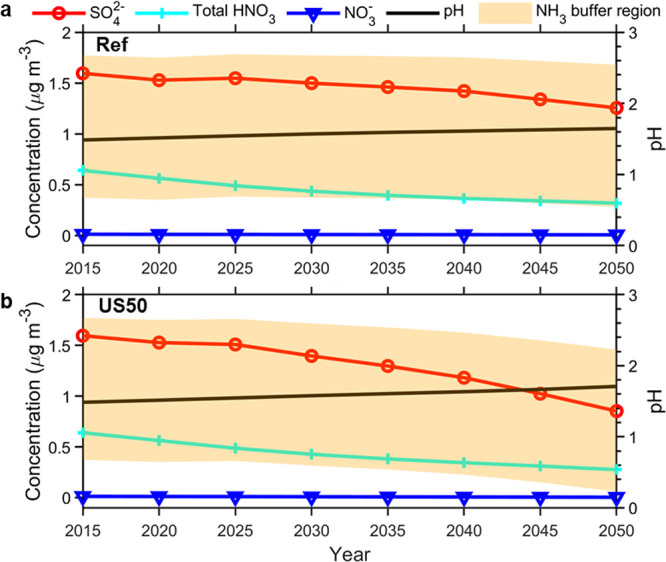
Predicted future U.S. pH trends. (a) “Ref” scenario is based on Shi et al.,^31^ while (b) “US50” scenario is more stringent as assumed in Ou et al.^32^ The shaded area indicates the NH_3_ buffered pH ranges (i.e., the ammonia p*K*_a_^*,ni^ ± 1).

We further did a rough estimate of future aerosol pH variation trends in SE-US. We applied two scenarios, the “Ref” scenario based on the prediction of the GCAM-USA model following the work of Shi et al.,^31^ while the “U50” scenario is a more stringent scenario, assuming 50% reduction target is applied in 2050 compared to the Ref scenario.^32^ We assume the reduction of sulfate and total nitrate is in proportional to the SO_2_ and NOx reduction percentages compared to 2015, while other species are kept constant. As shown in Figure 5, under both scenarios, SE-US aerosol acidity is predicted to remain in the ammonia-buffered regime and will increase only slightly, and nitrate will remain largely in the gas phase, in agreement with the conclusion of Weber et al.^10^

The above analysis shows how the multiphase buffer theory can be applied to explain the long-term trends in aerosol acidity against changes in aerosol compositions. The stable pH in the past and as projected in the future illustrated the strong capacity of ammonia in buffering aerosol acidity and the secondary inorganic aerosol compositions against stringent reductions of acidic gases in the U.S. As the SE-US is projected to remain in the ammonia-buffered regime, the sulfur emission controls are projected to result in effective sulfate reductions, without causing higher particulate nitrate concentrations.

## Supporting Information Available

Supplementary Figures S1–S4 showing model validation and comparisons and the influence of mixing states on the ammonium-to-sulfate ratios (PDF)
