# Supplementary material for: Revisiting the Key Driving Processes of the Decadal Trend of Aerosol Acidity in the U.S
Source: ACS Environ Au. 2022 May 6;2(4):346–53. doi: 10.1021/acsenvironau.1c00055 (PMC10125332; doi:10.1021/acsenvironau.1c00055)
Supplement: Supplementary file 1 — vg1c00055_si_001.pdf [file vg1c00055_si_001.pdf]

Supporting Information for

Revisiting the key driving processes of the decadal trend of aerosol acidity in the U.S.

Guangjie Zheng[1], Hang Su[2], Yafang Cheng[1*]

[1] Minerva Research Group, Max Planck Institute for Chemistry, Mainz 55128, Germany

[2] Multiphase Chemistry Department, Max Planck Institute for Chemistry, Mainz 55128, Germany

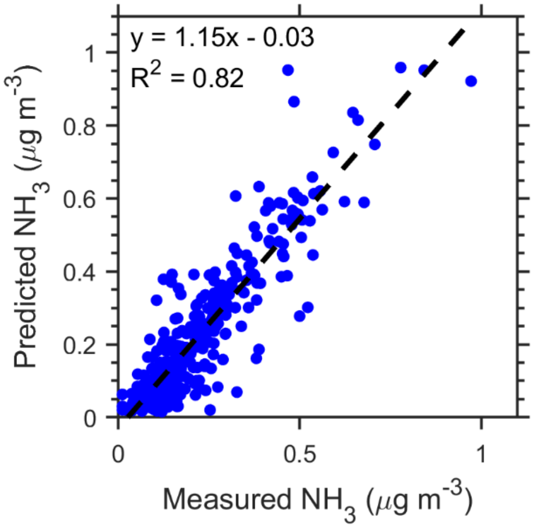

Fig. S1 Evaluation of the ISORROPIA thermodynamic model through comparison of the model predicted NH_3(g) concentrations against the measured ones. Data are based on the hourly measurements in SEARCH CTR site in summer 2004 to 2016, when NH_3(g) measurements are available.

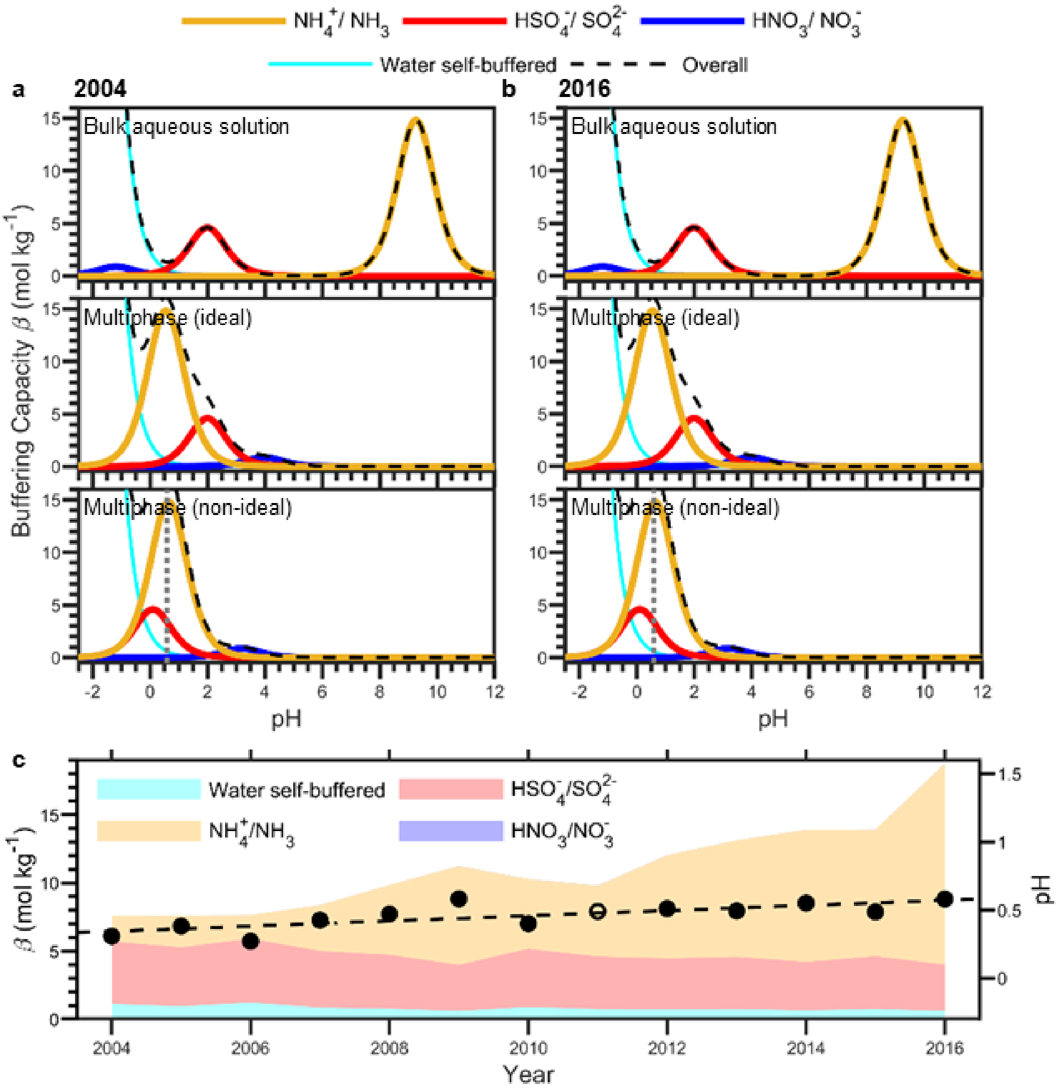

Fig. S2 Same as Fig. 2, but calculated with the E-AIM model.

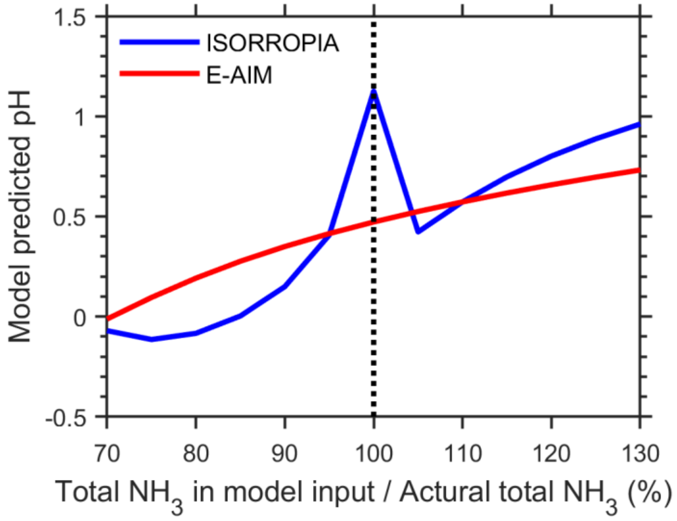

Fig. S3 Sensitivity of the pH predicted by ISORROPIA and E-AIM models with the input total NH_3 in SE-US in summer 2008. Data are based on the average compositions in SEARCH CTR site in summer 2008, with the average RH of 74% and temperature of 298 K. Only sulfate, nitrate and total ammonias are considered here. When the total NH_3 in model input is varied by 100% ± 30% of the measured ones, the E-AIM predicted pH show smooth variations, while the ISORROPIA predicted pH show a sudden jump when the input total NH_3 levels are within ± 5% of the measured ones. This is likely related to minor bugs in the ISORROPIA algorithm.

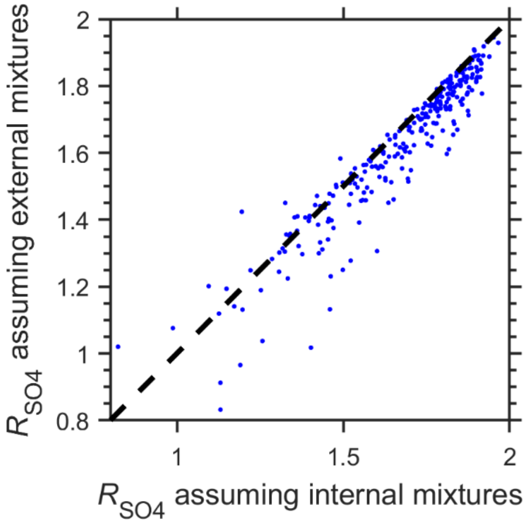

Fig. S4 Influence of mixing states on the R_{SO4}, where R_{SO4} = $([NH_4^+]-[NO_3^-])/[SO_4^{2-}]_{tot}$. Here the RSO4 are calculated from ISORROPIA predictions with measurements in SEARCH-CTR site in summer 2008 to 2016. For the internal mixture scenario, the inputs are total observed $PM_{2.5}$ chemical compositions and gas precursors. For the fully external mixing scenario, NVCs and NO_3^- are assumed to reside preferentially in the coarse mode which is partially included in $PM_{2.5}$ [1], and won't influence the gas-particle partitioning of the Aitken/Accumulation modes dominated by SO_4^{2-}, NH_4^+, and NO_3^-. In this case, NVCs are assumed to be fully neutralized first by available total HNO_3 and then total HCl, while the sulfate is assumed to be combined with NVCs only when the equivalent charge molar concentrations (in μeq m^{-3}) is larger than that of total HNO_3 and HCl. The coarse mode aerosol compositions are deducted from the measured $PM_{2.5}$ chemical compositions in the input of thermodynamic models.

REFERENCES

1. Seinfeld, J. H.; Pandis, S. N., *Atmospheric chemistry and physics: from air pollution to climate change*. John Wiley & Sons: 2016.
